# Alcohol-Induced Sleepiness and Memory Function

**Published:** 1995

**Authors:** Timothy Roehrs, Thomas Roth

**Affiliations:** Timothy Roehrs, Ph.D., is director of research at the Sleep Disorders and Research Center of the Henry Ford Hospital and adjunct professor of psychiatry at Wayne State University, Detroit, Michigan. Thomas Roth, Ph.D., is division head of the Sleep Disorders and Research Center of the Henry Ford Hospital, Detroit, Michigan, and adjunct professor of psychiatry at the University of Michigan, Ann Arbor, Michigan

**Keywords:** sedatives, AODE (alcohol and other drug effects), sleep, AOD impairment, neurotransmitters

## Abstract

Alcohol has sedative, as well as performance and memory-impairing effects. Several independent lines of research indicate that alcohol-induced sleepiness may contribute to the observed memory and performance impairment. Such a link would imply that alcohol consumption in combination with other drugs or conditions that enhance sleepiness could increase the risk for alcohol-related impairment.

Alcohol is known to impair various aspects of cognitive functioning, including learning and memory (Birnbaum and Parker 1977). Alcohol-induced memory impairment has been studied extensively both by researchers and clinicians, partly because memory impairment has everyday practical consequences for the affected patient. In addition, precise methods exist to assess memory functions. However, despite the large number of studies on alcohol-induced memory impairment, there is little consensus about the specific components of memory affected by alcohol or about the neurobiological mechanisms underlying alcohol’s effects.

A useful method of conceptualizing the way alcohol may affect memory is offered by Curran (1991). This concept entails that mood, level of sleepiness/ alertness (or, as Curran describes it, arousal), and memory all are interrelated. Alcohol is known to affect directly each of these factors. And by acting on one factor, alcohol also can indirectly affect the other factors because of their interrelations.

This article reviews a wide range of research findings contributing to the hypothesis that alcohol’s direct sedative effects (i.e., alcohol-induced sleepiness)—in addition to its direct memory effects—contributes to alcohol’s amnestic (i.e., memory impairing) effects. Studies on both healthy, sleep-deprived people and on patients with sleep disorders and studies of the effects of sedative drugs provide the basis for this hypothesis. Studies separately assessing alcohol’s sedative and amnestic effects or simultaneously measuring alcohol’s sedative and performance-disruptive effects provide further support. The article also describes some of the neurotransmitter systems controlling sleep and wakefulness that are affected by alcohol and other sedative drugs and that may underlie the association of sedation and memory impairment. Finally, some important practical implications of the potential correlation between alcohol’s sedative and memory-impairing effects are discussed.

## What Is Sleepiness?

Like hunger and thirst, sleepiness is considered a basic physiological drive state. It reflects the organism’s need or pressure for sleep. Like other physiological drive states, the level of sleepiness is difficult to assess. Despite a general tendency toward increasing sleepiness after sleep loss, most sleep deprivation studies find some inconsistencies in the subjects’ personal assessment of how sleepy they are (Monk 1991; Roth et al. 1994).

Research has shown that people’s ability to accurately judge their degree of sleepiness depends on several factors, such as internal point of reference, environmental demands, and time of day (Roth et al. 1994). For example, a person not getting enough sleep for an extended period will lose the internal reference to the experience of full alertness and therefore may underestimate his or her level of sleepiness. Similarly, people often judge their level of sleepiness to be higher in boring, nonstimulating situations in which environmental demands to stay alert or to pay attention are reduced. Finally, most people experience a circadian fluctuation with increased sleepiness over the midday and increased alertness in the early evening.

To assess sleepiness or alertness or the sedative effects of drugs, such as alcohol, scientists have asked people to self-rate their sleepiness or have used standard laboratory tests of performance. However, for the reasons stated above, self-ratings of sleepiness or sedative drug effects may be inaccurate (Roth et al. 1982). Similarly, performance tests sometimes are insensitive to the effects of small doses of sedative drugs or low breath alcohol concentrations.

A method to assess sleepiness objectively has been developed, however. This method conceptually is based on an observation originating in the 19th century that as sleep loss progresses over time, people increasingly experience uncontrollable brief naps or microsleeps (Patrick and Gilbert 1896). In the Multiple Sleep Latency Test (MSLT), researchers quantify sleepiness by giving subjects repeated opportunities to fall asleep (Carskadon et al. 1986; Roth et al. 1994). Typically, four to five tests are conducted at 2-hour intervals in a sleep-conducive environment. Physiological recordings of the subject’s brain waves and eye movements determine the exact moment of sleep onset. The time between lying down and sleep onset (i.e., the latency) is a measure of the subject’s level of sleepiness.

**Figure 1:**
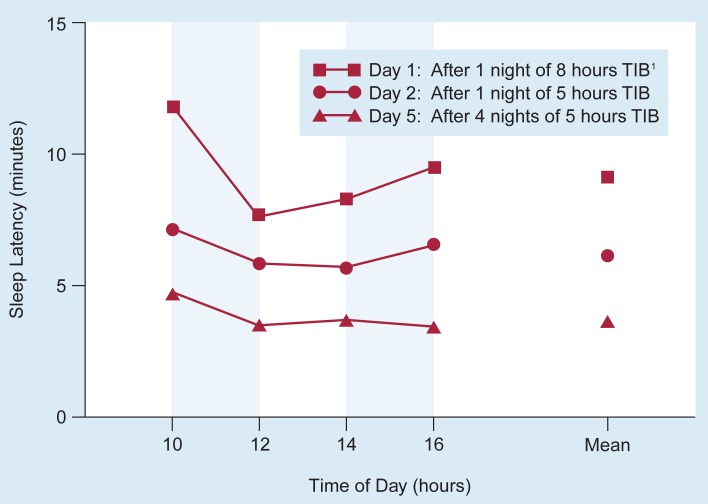
Sleepiness increases and sleep latency (i.e., the time [in minutes] between lying down and the onset of sleep) decreases after progressive sleep restriction. Subjects were tested with the Multiple Sleep Latency Test on days 1, 2, and 5. On the test days, subjects’ sleep latency was determined four times. The figure shows both the sleep latency at each measurement and the mean of all four measurements. ^1^TIB = time in bed. SOURCE: Adapted from Zwyghuizen-Doorenbos et al. 1988.

People with a high degree of sleepiness (e.g., because they are totally deprived of sleep, have had insufficient sleep relative to their biological needs, or are suffering from sleep disorders) fall asleep rapidly when given the opportunity to sleep. These people have a short latency on the MSLT. For example, limiting sleeping time by limiting time-in-bed (TIB) to 5 hours for several consecutive nights progressively decreases the subjects’ sleep latency over the test days (figure 1; Zwyghuizen-Doorenbos et al. 1988). In people with longer TIB’s or people who are treated successfully for a sleep disorder, sleep latency increases on the MSLT (i.e., they are more alert).

The MSLT also can measure the effects of stimulant and depressant drugs on sleepiness (Roth et al. 1994). Stimulant drugs increase and depressant drugs decrease sleep latency in a dose-dependent manner. For example, increasing doses of depressant drugs lead to systematic increases in sleepiness as measured by the MSLT. Thus, the reliability and validity of the MSLT have been established under different experimental conditions (Roth et al. 1994).

## Sleepiness and Memory Impairment

A correlation between sleepiness and memory impairment already was observed in the last century when Patrick and Gilbert (1896) reported that after 72 hours of wake-fulness, subjects were unable “to attend to a memory task.” Subsequent systematic studies of sleep deprivation and sleep restriction in healthy people supported the association of sleep loss with memory loss (Dinges and Kribbs 1991).

MSLT studies of patients with different sleep disorders have helped to confirm that sleepiness, which is the consequence of sleep loss or sleep fragmentation (i.e., frequent sleep interruptions), is the intervening variable causing the memory impairment:

- Patients with sleep apnea syndrome stop breathing during their sleep and wake up to resume breathing, leading to sleep fragmentation. Standardized tests of neuropsychological functioning in these patients detect impairment on a range of cognitive functions, including memory (Roehrs et al. in press). The extent of sleep fragmentation at night and the resulting daytime sleepiness directly correlate with the extent of neuropsychological impairment (Roehrs et al. in press).
- Patients with narcolepsy, a sleep disorder with unknown causes, suffer from excessive daytime sleepiness as documented by short MSLT latencies. Fifty percent of the patients have memory lapses, and 80 percent of the patients report episodes of automatic behavior (i.e., an ongoing behavioral activity that the patient does not remember doing, such as missing freeway exits, driving through stop signs, or writing nonsense) (Aldrich 1992).
- Patients with chronic sleep restriction (i.e., their sleep is normal but their TIB is reduced by 1 to 2 hours per night relative to their biological needs) complain about persistent daytime sleepiness and have reduced sleep latency on the MSLT (Roehrs et al. 1983). Merrion and colleagues (1992) found that these patients also showed impairment on standardized neuropsychological tests, specifically on memory tests.

Memory development encompasses several processes, or phases, that occur when information is stored in the brain. One old and rather simplistic—but in this context, sufficient—model of memory processing distinguishes three phases of memory development (Lister et al. 1987). The first phase is the stimulus registration or acquisition phase, in which information is entered into short-term memory. The second phase is the consolidation of information from short-term into long-term memory (i.e., memory for more than 30 seconds). The third phase includes the retrieval of information from long-term memory.

Sleepiness can interfere with all three phases of memory development. Elkin and Murray (1974) assessed sleepiness effects on the acquisition phase by studying sleep-deprived subjects who experienced uncontrollable microsleeps (i.e., sleep episodes of less than 15 seconds). During microsleeps, the subjects did not respond to a given stimulus, suggesting that they did not register the stimulus.

Other studies found that the process of sleep onset or increasing sleepiness also disrupts memory consolidation. In a study by Guilleminault and Dement (1977), subjects were presented with a stimulus at 1-minute intervals as they were falling asleep. Even when stimulus registration was ascertained, the subjects’ memory when they were awakened 10 minutes later decreased as the stimulus occurred closer to sleep onset. Other studies found a general slowing of cognitive functions as a consequence of sleep loss (Dinges and Kribbs 1991). This may reflect a reduced information consolidation or impaired retrieval of information from long-term memory.

## Sedating Drugs and Memory Impairment

The correlation between sleepiness and memory impairment has been established not only in sleep deprivation studies and in patients with sleep disorders but also in studies of the effects of sedative drugs. Because alcohol also is a sedative drug, these studies may help elucidate the mechanisms of alcohol’s actions on the brain.

Sedative drugs interact with several different neurotransmitter^1^ systems and neurochemicals (i.e., neuropeptides) of the brain that may be involved in regulating sleep and wakefulness and are also involved in memory and learning (Lister et al. 1987; Jones 1994). It is beyond the scope of this article to describe all the systems and neurochemicals that might be involved; it is a very complex body of research. However, some systems targeted by commonly used drugs that have both sedative and memory-impairing effects will be mentioned, owing to some important practical implications discussed later in the article.

### GABA and GABA Agonists

Gamma-aminobutyric acid (GABA) is one of the major inhibitory neurotransmitters and may promote sleep (Jones 1994). Some sedative drugs, such as benzodiazepines and barbiturates, are GABA agonists (i.e., they mimic or facilitate GABA-mediated inhibition of adjacent cells).

GABA agonists also have amnestic effects (Lister 1985) that parallel their sedative effects as determined by MSLT (Roth et al. 1990; Roehrs et al. 1994*b*). Findings that a drug called flumazenil can reverse both benzodiazepine-induced sedation and amnesia (Dorow et al. 1987) support a correlation between sedative and amnestic effects. In contrast, Hommer and colleagues (1993) reported that the two effects are independent. This lack of a sedative-amnestic correlation, however, may be due to the more inconsistent, self-report-based assessment of sedation used in their study.

GABA agonists can interfere with both the stimulus registration phase and the consolidation phase of memory development (Lister 1985; Roth et al. 1990). The extent to which these drugs interfere with memory retrieval is unclear (Roth et al. 1990).

### Acetylcholine and Acetylcholine Antagonists

Acetylcholine is a neurotransmitter that is important for maintaining wakefulness and for increasing the firing rate of nerve cells in the cortex. Drugs that are acetylcholine antagonists (i.e., that counteract acetylcholine activity) have both sedative and memory-impairing effects, as do the GABA agonists (Preston et al. 1989). For example, the acetylcholine antagonists scopolamine and atropine, which are common in over-the-counter medications and are used to treat respiratory and gastrointestinal disorders, can disrupt episodic and semantic memory functions (Higgins et al. 1989; Preston et al. 1989). These effects on memory are accompanied by increased sleepiness as determined through self-reports.

A role of acetylcholine and acetylcholine antagonists in controlling memory functions is supported by the so-called cholinergic hypothesis of dementia. This hypothesis suggests that dementia is caused by the degeneration and dysfunction of cholinergic (i.e., acetylcholine-using) neurons. Administration of acetylcholine antagonists would correspond to a cholinergic dysfunction.

### Histamine and Histamine Antagonists

Histamine also is a neurotransmitter promoting wakefulness. Histamine antagonists, also called antihistamines, are used for symptomatic treatment of common colds, hay fever, and allergies and are the most common ingredients in over-the-counter sleep medications. Antihistamines that readily cross the blood-brain barrier at clinical doses also increase daytime sleepiness in a dose-dependent manner as measured both subjectively and objectively with the MSLT (Nicholson and Stone 1986).

Many studies assessing the effects of antihistamines on psychomotor performance detect impaired performance on the tests (Roth et al. 1987). The time course and dose dependence of the performance-disruptive effects parallel the time course and dose dependence of the antihistamine’s sedative effects. The tests used in these studies did not specifically assess cognitive functioning and memory impairment. However, many of the studies demonstrated performance impairment on the digit-symbol substitution test,^2^ which requires some memory abilities.

These examples show that drugs affecting various neurotransmitter systems can be associated with both sedative and amnestic effects. Some researchers have attempted to reverse the sedative and amnestic effects of a drug affecting one neurotransmitter system by using a drug affecting another neurotransmitter system (Preston et al. 1989). These so-called cross-reversal experiments were not successful, indicating that the different neurotransmitter systems are independent and cannot compensate for each other. However, the lack of cross-reversal between agonists and antagonists of different neurotransmitters does not invalidate the general hypothesis of a correlation between sedative and amnestic drug effects.

## Alcohol-Induced Sedation and Memory Impairment

### Alcohol’s Sedative Effects

Laboratory studies evaluating alcohol’s stimulating and sedative effects have found a biphasic response by the test subjects (Pohorecky 1977). At low alcohol doses and while the blood alcohol concentration (BAC) is ascending, alcohol’s stimulating effects prevail. In contrast, at high alcohol doses and while the BAC is descending, alcohol primarily has sedative effects. Recently, Petrucelli and colleagues (1994) confirmed the biphasic effects of alcohol using the MSLT method. An alerting effect (i.e., increased sleep latency) was found over the first hour during the ascending phase of the BAC curve and at peak alcohol concentration; subsequently, a sedating effect (i.e., decreased latency) was observed.

**Figure 2:**
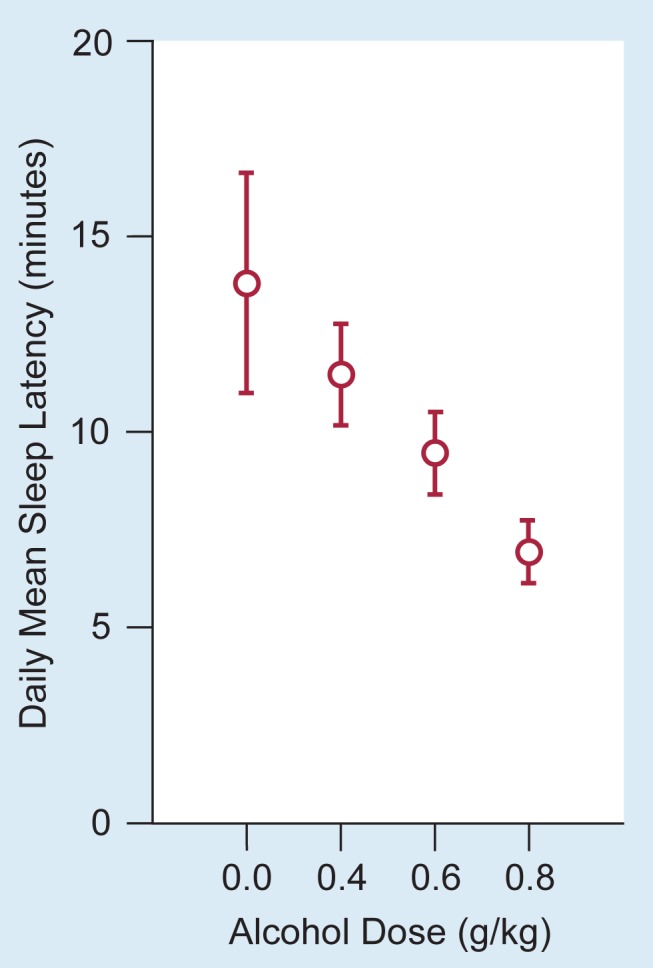
Alcohol consumption reduces sleep latency (i.e., increases sleepiness) in a dose-dependent manner. Sleep latency (in minutes) was determined after the subjects received alcohol doses of 0.0, 0.4, 0.6, and 0.8 gram per kilogram of body weight. The circles represent the mean values of four tests over the course of a day. Vertical lines indicate the standard error. SOURCE: Adapted from Roth et al. 1989/1990.

Other studies have focused on alcohol’s sedative effects throughout the descending phase of the BAC curve and beyond. Alcohol’s sedative effects as measured by the MSLT are dose dependent (figure 2; Roehrs et al. 1989/1990; Zwyghuizen-Doorenbos et al. 1988). With increasing amounts of alcohol (the doses are equivalent to two to six beers), sleep latency decreases drastically, indicating an increasing sedative effect. Furthermore, sedation continues for at least 2 hours after the BAC has returned to 0 (Roth et al. 1989/1990).

### Alcohol’s Memory-Impairing Effects

Alcohol’s amnestic effects have been studied extensively. Birnbaum and Parker (1977) found that the degree of amnesia increased with larger doses of alcohol. Most studies report that alcohol impairs the acquisition of new information but does not affect the retrieval of previously memorized information (Mungas et al. 1994). The acquisition impairment occurs both at the attention phase and at the consolidation phase of memory processing. This amnestic effect has been described as a failure to process information “deeply” or as a “slowing of the processing rate” (Mungas et al. 1994; Rabbitt and Maylor 1991). Alcohol affects several memory systems. Semantic memory and episodic memory clearly are altered (Mungas et al. 1994). In addition, there are indications that perceptual memory is impaired (Mungas et al. 1994).

### The Link Between Alcohol-Induced Sedation and Amnesia

So far, no studies have established the correlation between alcohol’s sedative and amnestic effects by simultaneously assessing memory impairment and objective sleepiness (e.g., with the MSLT). However, several studies provide indirect evidence for such a correlation.

Roehrs and colleagues (1989) simultaneously studied alcohol’s sedative and performance-disruptive effects. The subjects received 0.75 gram of alcohol per kilogram of body weight. The sedative effects of this alcohol dose were measured by the MSLT. Performance-disruptive effects were assessed in two ways: (1) with a divided attention task, in which subjects tracked a moving target on a video screen while simultaneously responding to other stimuli appearing on the screen, and (2) with an auditory vigilance task, in which subjects detected long tones against the background of shorter tones. The subjects responded more slowly on both tasks but did not omit any responses, suggesting that alcohol leads to the cognitive slowing described in the sleep loss literature. Subjects with a higher degree of sleepiness tended to show a higher degree of performance impairment. The alcohol effects observed on a divided attention task or an auditory vigilance task likely predict a memory-impairing effect. This is suggested by studies of the sedative effects of different benzodiazepines that included divided attention and auditory vigilance tasks as well as memory tasks (Roehrs et al. 1994*b*).

Researchers also have measured alcohol-induced memory impairment and self-rated sleepiness. Rabbitt and Maylor (1991) found comparable dose dependence for both measures: The increase in memory impairment after larger alcohol doses was paralleled by a similar increase in sedation. The researchers obtained similar results when they analyzed the effects of the benzodiazepine triazolam. Other studies, in contrast, suggest that alcohol’s amnestic and sedative effects are independent of each other (Lister et al. 1987). Some of the inconsistencies among the studies may be due to the relatively unreliable self-reports used to assess sedation. Therefore, studies objectively measuring alcohol-induced sedation and concurrently assessing alcohol’s amnestic effects are needed to resolve these discrepancies.

### Preexisting Sleepiness and Alcohol-Induced Performance Impairment

Another strategy to establish a link between sleepiness and performance impairment—and, by inference, memory impairment—is to first manipulate subjects’ level of sleepiness by reducing or extending their TIB for one or more nights. The subjects then receive alcohol, and alcohol’s sedative and performance-disruptive effects are assessed (Zwyghuizen-Doorenbos et al. 1988; Roehrs et al. 1989, 1994*a*).

Reducing TIB increases the level of sleepiness the following day; the increased sleepiness enhances the sedative and performance-disruptive effects of alcohol (Zwyghuizen-Doorenbos et al. 1988). For example, an alcohol dose of 0.4 gram per kilogram of body weight has a lower sedative effect than an alcohol dose of 0.8 gram per kilogram of body weight if all subjects have had the same TIB the previous night. However, if subjects receiving the low alcohol dose have had only 5 hours TIB for 5 consecutive days and subjects receiving the high alcohol dose have had 8 hours TIB, both alcohol doses have the same sedative effect.

In a similar experiment, simulated driving and psychomotor performance were assessed in subjects who received an alcohol dose of 0.6 gram per kilogram of body weight producing breath ethanol concentrations of 50 milligram-percent (i.e., half the legal intoxication level in most States) and who had either 8 or 4 hours TIB (Roehrs et al. 1994*a*). Subjects with 4 hours TIB exhibited significantly greater impairment than subjects with 8 hours TIB. The increased alcohol effect was not due to differences in alcohol metabolism after reduced TIB, because breath alcohol levels were not affected by the TIB manipulation (Zwyghuizen-Doorenbos et al. 1988; Roehrs et al. 1994*a*).

In contrast, longer TIB reduces the level of sleepiness (i.e., increases alertness) and leads to an attenuation of some of alcohol’s effects (Roehrs et al. 1989). Subjects receiving an alcohol dose of 0.75 gram per kilogram of body weight after 8 hours TIB exhibited sedation and performance impairment, compared with subjects receiving a placebo after 8 hours TIB. However, after 7 nights of 10 hours TIB, the subjects experienced no effects from the same alcohol dose. Again, breath alcohol levels did not change as a result of the TIB manipulation.

### Impact of Alertness-Enhancing Measures

Alcohol’s sedative and performance-disruptive effects can be attenuated by enhancing the basal level of alertness after alcohol consumption, for example, with daytime naps. Roehrs and colleagues (1989/1990) assessed the effects of a 0.5 gram per kilogram of body weight alcohol dose after the subjects did or did not have a 60-minute nap. The nap completely reversed the sedative effect and attenuated the performance-disruptive effects of the alcohol.

**Figure 3:**
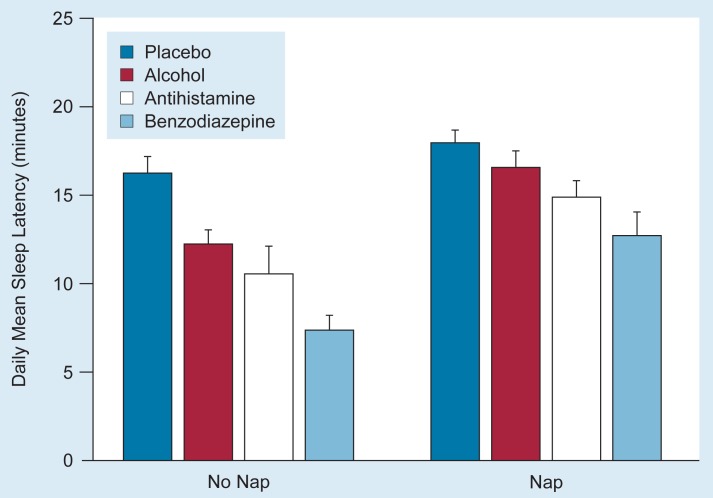
Different sedative drugs differentially decrease sleep latency (i.e., increase sleepiness). All subjects were tested under four conditions: after receiving a placebo, after receiving alcohol (0.6 gram per kilogram of body weight), after receiving the antihistamine diphenhydramine (50 milligrams), and after receiving the benzodiazepine triazolam (0.25 milligram). For each condition, the subjects were tested over a 2-day period. On one day, they took a 1-hour nap 1 hour after drug administration; on the other day, they did not take a nap. The sleep latency for the different conditions is the mean of four measurements taken over the course of the day. Vertical lines indicate the standard error. SOURCE: Adapted from Roehrs et al. 1993.

Another study examined the capacity of a 60-minute nap to reverse the sedative and performance-disruptive effects of alcohol, a benzodiazepine, and an antihistamine (Roehrs et al. 1993). The effectiveness of the nap to reverse the sedative effects of the drugs tested was inversely related to the extent of sedation initially produced by the drugs (figure 3). For example, the benzodiazepine had the strongest sedative effect, which was least reversible by the nap. Alcohol, in contrast, had the smallest sedative effect. This effect was almost completely reversed by the nap.

It is important to note that performance in these studies was assessed using the divided attention and vigilance tasks and therefore did not directly measure memory impairment. Although a thorough evaluation of alcohol-induced sedation and amnesia is needed in the future, the available studies support the hypothesis of a correlation between sedative and amnestic alcohol effects for at least two reasons. First, the alcohol doses used in these studies produced sedation levels (as measured by MSLT) comparable with the levels achieved in some of the sedative drug studies that demonstrated amnestic drug effects. Second, the alcohol doses used to assess objectively measured sedation and performance disruption are comparable with those used in studies that demonstrated alcohol’s amnestic effects without objective measures of sedation levels.

## Mechanisms of Alcohol-Induced Sedation and Amnesia

Alcohol affects some neurotransmitter systems in the same way that sedative drugs do. For example, alcohol facilitates GABA-mediated inhibition (i.e., acts as a GABA agonist) and reduces the release of acetylcholine (i.e., acts as an acetylcholine antagonist) (Hoffman and Tabakoff 1985). Consequently, alcohol could mimic the actions of other sedative drugs. Alcohol additionally affects two other neurotransmitters that regulate sleep and wakefulness. One is serotonin, a neuromodulator of sleep; the other is glutamate, an excitatory neurotransmitter promoting wakefulness. Interaction with these neurotransmitter systems may contribute to alcohol’s sedative effects. The contributions of serotonin and glutamate to memory functions currently are being studied extensively.

## Conclusions and Implications

Independent experiments have shown that alcohol causes memory impairment and that alcohol causes sedation. This article has reviewed information suggesting that the two effects may be linked, that is, that alcohol’s amnestic effects are related to its sedative effects. Evidence supporting this hypothesis comes from sleep deprivation studies in healthy people, studies of patients with sleep disorders, studies of drugs with sedative effects, and studies of the interaction between alcohol’s sedative and performance-disruptive effects. However, the sleepiness-memory hypothesis of alcohol effects has not yet been tested directly by manipulating levels of sleepiness and objectively measuring sedation and memory impairment.

An association between alcohol’s sedative and amnestic effects could have practical implications for both research and clinical issues. For researchers, such an interaction could affect the design of experiments assessing alcohol’s amnestic or performance-disruptive effects. For example, researchers would need to control for, or at least document, the amount of sleep their subjects get before the experiment. Similarly, the time of day at which tests are performed could affect study results, given the normal variations in people’s sleepiness levels across the day. Inattention to these influences could distort study results and cause inconsistencies when comparing results between studies.

From a clinical standpoint, the interaction between alcohol’s sedative and amnestic effects would imply that alcohol consumption combined with any condition or drug producing sleepiness could increase the risk for alcohol-induced memory impairment. For example, subgroups of the general population are known to be sleepier than average. People who shift their sleep schedule frequently (e.g., night workers or shift workers) are much sleepier than are people with a regular nighttime sleep schedule. Older people, who experience more fragmented sleep and who are more likely to have undetected sleep disorders, are sleepier than are younger people. In these risk groups, lower alcohol doses than predicted could induce memory impairment. Younger people who periodically sleep less (e.g., when studying for exams) similarly may experience memory problems after consuming alcohol in amounts they usually tolerate.

In addition, drugs with sedative effects could lead to amnesia when combined with alcohol, even at doses normally considered safe. Benzodiazepines, which often are used for patients undergoing alcoholism treatment, could contribute to amnesia if the patients relapse. A brief report in the late 1980’s described three clinical cases of global amnesia (i.e., total amnesia for recent events) associated with the concurrent use of the benzodiazepine triazolam and alcohol (Morris and Estes 1987). Many over-the-counter cold medications and many antidepressants have anticholinergic and/or antihistaminic ingredients that could contribute to amnesia after alcohol consumption, even in social drinkers. In addition, recovering alcoholics with coexisting depression often are treated with antidepressants. If a relapse occurs, the sedating side effects of these medications could increase the risk for amnesia.

## Footnotes

1 For a definition of this and other technical terms, see the glossary on pp. 136–137.

2 For a description of this test, see the article by Evert and Oscar-Berman on pp. 89–96.
